# No evidence of increasing *Haemophilus influenzae* non-b infection in Australian Aboriginal children

**DOI:** 10.3402/ijch.v72i0.20992

**Published:** 2013-08-05

**Authors:** Robert I. Menzies, Peter Markey, Rowena Boyd, Ann P. Koehler, Peter B. McIntyre

**Affiliations:** 1 The National Centre for Immunisation Research and Surveillance of Vaccine Preventable Diseases (NCIRS), Sydney, New South Wales, Australia; 2 Discipline of Paediatrics and Child Health, The Children's Hospital at Westmead, and the University of Sydney, Sydney; 3 Centre for Disease Control, Northern Territory Department of Health, Darwin, NT, Australia; 4 Applied Epidemiology training program, Australian National University, Canberra, Australia; 5 Communicable Disease Control Branch, South Australian Department of Health and Ageing, Adelaide, SA, Australia

**Keywords:** Haemophilus influenza, *oceanic ancestry group*, *epidemiology*

## Abstract

**Background:**

High, or increasing, rates of invasive *Haemophilus influenzae* (Hi) type a disease have been reported from North American native children from circumpolar regions, raising the question of serotype replacement being driven by vaccination against Hi type b (Hib). Indigenous Australians from remote areas had high rates of invasive Hib disease in the past, comparable to those in North American Indigenous populations.

**Objective:**

Evaluate incidence rates of invasive Hi (overall and by serotype) in Indigenous Australian children over time.

**Design:**

Descriptive study of Hi incidence rates by serotype, in the Northern Territory (NT) and South Australia (SA) from 2001 to 2011. Comparison of NT data with a study that was conducted in the NT in 1985–1988, before Hib vaccine was introduced.

**Results:**

The average annual rate of invasive Hi type a (Hia) disease in Indigenous children aged <5 years was 11/100,000 population. Although the incidence of Hi infection in Indigenous children in 2001–2003 was lower than during 2004–2011, this may be due to changes in surveillance. No other trend over time in individual serotypes or total invasive Hi disease, in Indigenous or non-Indigenous people, was identified. Compared to 1985–1988, rates in 2001–2011 were lower in all serotype groupings, by 98% for Hib, 75% for Hia, 79% for other serotypes and 67% for non-typeable Hi.

**Conclusions:**

There is no evidence of increases in invasive disease due to Hia, other specific non-b types, or non-typeable Hi in Australian Indigenous children. These data suggest that the increase in Hia some time after the introduction of Hib vaccine, as seen in the North American Arctic Region, is not common to all populations with high pre-vaccine rates of invasive Hib disease. However, small case numbers and the lack of molecular subtyping and PCR confirmation of pre-vaccine results complicate comparisons with North American epidemiology.

Although the climate of Central and Northern Australia is very different to that in the circumpolar region, the Indigenous people of these 2 regions share many similarities. These include the social determinants that lead to ill-health, such as poor environmental living conditions, poverty and remoteness (1, 2). Consequently, remote Indigenous Australian and North American peoples have been afflicted with very high rates of respiratory disease, including invasive bacterial diseases, pneumonia and otitis media. In the case of invasive *Haemophilus influenzae* (Hi) and pneumococcal disease, this has included broader serotype distributions, making disease control by vaccination more challenging although still successful overall (3).

South Australia (SA) has a population of 1.6 million including 31,000 Aboriginal and Torres Strait Islander (henceforth referred to as “Indigenous”) people, and covers an area of 380,000 square miles. The population is concentrated in the capital city Adelaide (1.2 million). The population of the Northern Territory (NT) is much smaller at 235,000, including 70,000 Indigenous people. The capital Darwin has 128,000 residents. The NT covers an area of 521,000 square miles. The majority of the landmass covered by these 2 jurisdictions is classified by the Australian Bureau of Statistics as “very remote” (Fig. 1). The climate is subtropical in the north, arid with wide temperature fluctuations in the centre, and Mediterranean on the coastal fringe in the south. It includes most of central Australia, from where the highest rates of Hi type b (Hib) and pneumococcal disease in the world were recorded in Aboriginal children (4, 5).

**Fig. 1:**
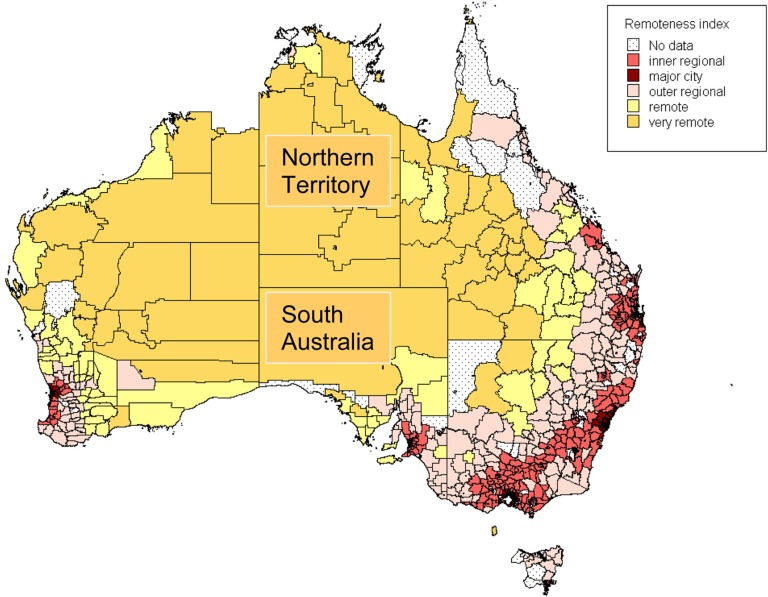
Australia by “remoteness classification”. *Source*: Australian Bureau of Statistics.

Hib vaccine was recommended and funded for all Australian infants from 1993, and since 2001, more than 90% of infants have completed the primary course by 12 months of age (6). In recent years, it has been suggested that the rates of invasive disease due to Hi type a (Hia) have been increasing in North American Indigenous children, particularly in the circumpolar region (7). Capsular switching of a virulence factor from serotype b to serotype a has also been demonstrated (8), and the possibility of a role of the Hib vaccine in causing serotype replacement has been discussed (9). Molecular testing has shown the predominance of a particular clone in Northern Canada and Alaska (10).

This study aims to examine rates of disease due to Hi, in particular, serotype a, in remote Australian Indigenous people, and to examine whether increases in Hia in the post-Hib vaccine era can be demonstrated in Australia.

## Methods

Invasive Hib infection has been reportable in SA and the NT since 1993. This was expanded to include all invasive Hi, by all public and private laboratories in the NT and by laboratories and medical practitioners in SA. Case reporting occurred as part of routine passive infectious disease surveillance. Although no formal audits have been conducted, surveillance coordinators (authors Peter Markey and Ann P. Koehler) consider surveillance processes to have been stable and satisfactory from 2001. Reporting is almost exclusively direct from laboratories to health authorities, with subsequent follow-up to medical practitioners. Clinical information is not collected.

Invasive Hi isolates from laboratories in these jurisdictions were referred for serotyping to IMVS Pathology in Adelaide or the Institute of Clinical and Pathological Medicine Research, Westmead (ICPMR), Sydney. Serotyping at ICPMR was done using serum coagglutination test kits: Phadebact *Haemophilus* Test (Bactus, Sweden) consisting of monovalent b antisera and polyvalent a, c–f antisera and Remel Hi agglutinating sera – monovalent a, c–f. Unusual or weak reactions were checked by PCR. In recent years, the PCR method was a multiplex that included an H (capsule-specific) target and type-specific genes for Hia and Hib. ICPMR laboratory was enrolled in the EU-IBIS Haemophilus Quality Assurance Program, Haemophilus Reference Laboratory, Respiratory & Systemic Infection Laboratory, Health Protection Agency, UK, until recently when the programme ended. Serotyping at IMVS was done using Remel serum agglutination test kits for monovalent b, polyvalent a, c–f and monovalent a, c–f. No molecular testing or QA programme was undertaken.

Incidence rates were calculated using population estimates for relevant years from the Australian Bureau of Statistics (11). Rates were compared using the rate ratio and 95% confidence intervals, calculated using the method of Rothman (12). The Chi-squared test was used for testing the significance of differences in proportions.

Average annual incidence rates in NT Indigenous children for 2001–2011 were compared with a study from the pre-vaccine era that covered the same geographic area (13). This was the only pre-vaccine study that provided disease rates on all Hi serotypes in Indigenous Australian children. It consisted of an audit of Hi cases occurring in children aged<5 years from all 5 hospital laboratories in the NT from 1985 to 1988, which included samples referred from parts of the central desert region of SA. There were 108 cases identified, 80 of which were in Indigenous children. Testing was conducted at multiple hospitals using Wellcogen (Wellcome Diagnostics, Dartford, England) latex agglutination or Phadebact serum coagglutination test kits for monovalent b. Non-type b isolates were forwarded to the IMVS laboratory in Adelaide (formerly Adelaide Children's Hospital) for serotyping using Wellcogen monovalent antisera.

## Results

There were a total of 238 cases of invasive Hi infection reported from 2001 to 2011 in the 2 jurisdictions, 86 from the NT and 152 from SA (Table I). Indigenous status was unknown in 13 cases, 12 of which were from SA.

**Table I:** Invasive *Haemophilus influenzae* disease by serotype, Indigenous status^a^ and age group

		Type a	Type b	Other	Nontypeable	Not typed	Total	Rate^b^

		No. (%)	No. (%)	No. (%)	No. (%)	No. (%)	No. (%)	
<5	Indigenous	13 (33)	12 (31)	2 (5)	10 (26)	2 (5)	39 (100)	24.8
	Non-Indigenous	0 (0)	9 (41)	3 (14)	8 (36)	2 (10)	22 (100)	2.0
5–10	Indigenous	0 (0)	1 (20)	2 (40)	2 (40)	0 (0)	5 (100)	6.8
	Non-Indigenous	0 (0)	1 (33)	0 (0)	2 (67)	0 (0)	3 (100)	0.3
11–60	Indigenous	4 (8)	8 (17)	4 (8)	26 (55)	5 (10)	47 (100)	6.2
	Non-Indigenous	0 (0)	1 (3)	5 (15)	24 (73)	4 (10)	33 (100)	0.3
>60	Indigenous	1 (20)	1 (20)	0 (0)	1 (20)	2 (40)	5 (100)	9.3
	Non-Indigenous	0 (0)	2 (3)	7 (10)	59 (83)	4 (5)	71 (100)	2.0
Total	Indigenous	18 (20)	22 (23)	8 (8)	39 (40)	9 (9)	96 (100)	9.0
	Non-Indigenous	0 (0)	13 (9)	15 (12)	92 (71)	10 (7)	129 (100)	0.7

a There were 13 cases with unknown Indigenous status.

b Average annual incidence rate of Hi per 100,000 population.

Annualised invasive Hi incidence rates for Indigenous people were 10.5/100,000 (74 cases) in the NT and 7.1 (22) in SA, which were not significantly different (rate ratio 1.5, 95% CI 0.9–2.5). There was also no significant difference between the 2 jurisdictions in rates for non-Indigenous people, which were 0.74 (12 cases) and 0.70 (117), respectively (rate ratio 1.1, 0.5–1.9).

Serotyping results were available for 215 (96%) isolates with known Indigenous status (Table I). Of cases in Indigenous children aged <5 years with a known serotype 10/32 (30%), 2/6 (33%) were type a in the NT and in SA, respectively. In non-Indigenous people, the most commonly identified capsular type was non-typeable, in both the NT 3/8 (38%) and in SA 89/111 (80%) and these proportions were significantly different (P=0.005). The data from here on were analysed for the 2 jurisdictions combined, as the serotype distribution was similar in the 2 jurisdictions for the group of primary interest (Indigenous children), while cases in non-Indigenous people were representative of an urban population, predominantly from Adelaide.

The rates of Hi infection were higher in Indigenous compared to non-Indigenous people (rate ratio 13.8, 95% CI 10.4–18.0). In both groups, rates were highest in the extremities of age. Serotype distributions differed between age groups and Indigenous status (Table I). Non-typeable Hi isolates were most commonly identified overall, making up 44% of isolates sent for typing from Indigenous and 77% from non-Indigenous people. However, in children <5 years of age, non-typeable types were less likely to be found (27 and 40% of cases in Indigenous and non-Indigenous children, respectively). Hia infection was limited entirely to Indigenous people ((18) cases and 14 from the NT), and of those, 13 (72%) were aged <5 years. The average annual Hia disease incidence rate for NT and SA indigenous children aged <5 years was 10.5/100,000 from 2001 to 2011.

**Fig. 2:**
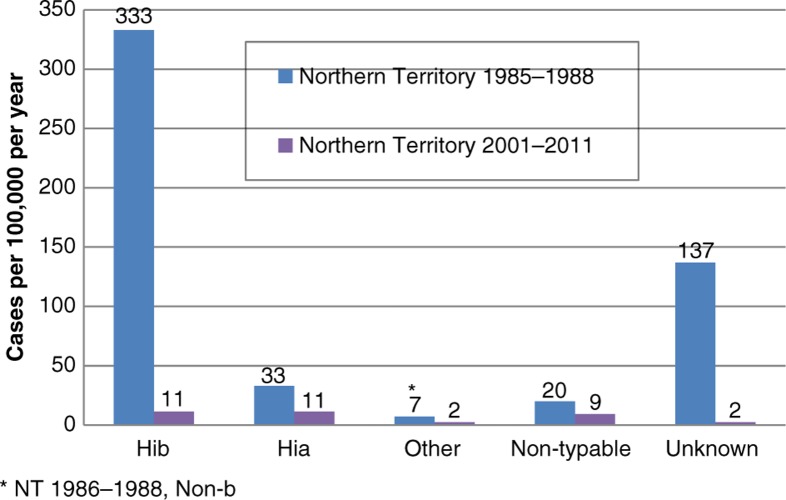
Invasive *Haemophilus influenzae* disease by serotype, Indigenous children <5 years.

Hi annual incidence rates from Indigenous children aged <5 years in the NT during 2001–2011 were much lower than that found during the period 1985–1988, with post-vaccination: pre-vaccination incidence rate ratios 0.03 (0.07–0.01) for Hib, 0.27 (0.11–1.25) for Hia, 0.28 for other encapsulated types and 0.45 for non-typeable isolates (Fig. 2; 95% CI for non-a and non-b types combined 0.13–1.40). Serotype data were available for a smaller proportion of isolates from the earlier study (74%), compared to recent NT reported cases in Indigenous children (95%). Adjustment for unknown serotype, by attributing unknown serotypes in proportion to the distribution of known serotypes, resulted in post- to pre-vaccination incidence rate ratios being 0.02, 0.25, 0.21 and 0.33 for Hib, Hia, other encapsulated and non-typeable isolates, respectively.

**Fig. 3:**
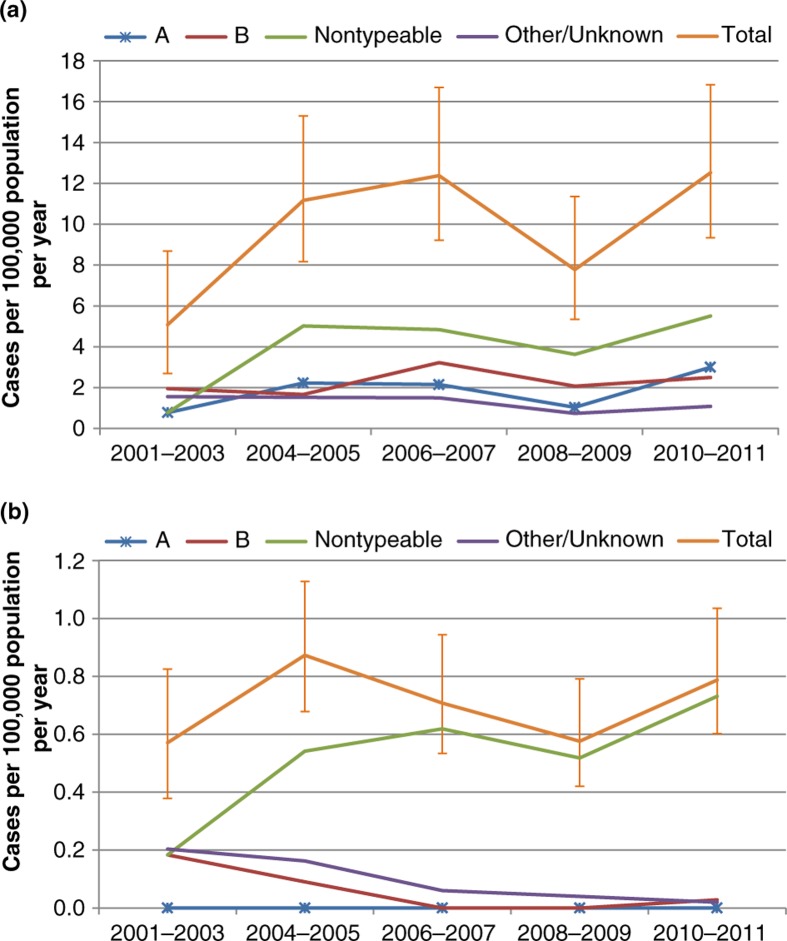
Incidence of invasive *Haemophilus influenzae* disease, Northern Territory and South Australia, 2000–2011. (a) Indigenous; (b) non-Indigenous.

Incidence rates by year are shown in Fig. 3, with years grouped to improve precision. Hi rates in Indigenous people of all ages were generally lower in 2001–2003 compared to 2004–2011, for both encapsulated and non-typeable types. These differences were significant for all Hi combined (rate ratio 0.5, 0.3–0.9), and non-typeable Hi (0.2, 0.02–0.7), but not for Hia (0.3, 0.04–1.4). In non-Indigenous people, there was no evident trend over time in total Hi, but an apparent increase in non-typeable Hi over time was accompanied by apparent decreases in serotype b and other/unknown types.

## Discussion

This study shows no statistically significant trends in incidence rates of Hia between 2001 and 2011 in the NT and SA Indigenous people. Rates of invasive Hi in Indigenous people were lower in the 2001–2003 period compared to 2005–2011, but differences were not significant for individual types. The average annual incidence rate for Hia in Indigenous children aged <5 years from 2001 to 2011 was 11/100,000 in the NT and 7 in SA. These rates were much lower than what was found in an audit of NT laboratories in 1985–1988 (33) (13). As expected, rates of Hib were much lower in 2001–2011 compared to 1985–1988, following the commencement of widespread vaccination in 1993. In fact, the difference (98% lower rates in the post-vaccination period) is similar to the estimated reduction in other populations following Hib vaccination (14, 15). However, rates for non-typeable Hi and other encapsulated serotypes were also substantially lower in the post-vaccination period, although by lesser amounts: 75, 79 and 67% lower for Hia, other encapsulated types and non-typeable Hi, respectively. Numbers of non-b cases were lower and the decreases were not statistically significant.

Possible explanations for lower rate point estimates in more recent times include under-ascertainment of cases in the post-vaccination period, general improvements in disease rates due to improved living conditions, changes in diagnostic practice, and false-positive results in the pre-vaccine era.

It is possible that the hospital laboratory audit in 1985–1988 detected a larger proportion of diagnosed cases than the passive laboratory reporting in place from 2001 to 2011. In fact, the lower rates of Hi in 2001–2003 may be related to under-ascertainment of cases in the early years of surveillance. However, the size of the decrease in Hib disease (98%) is similar to that attributed to vaccination in other settings (14). Any decreased sensitivity in the later study would be expected to be equally distributed across serotypes. Therefore, any real increase in Hia disease relative to other serotypes, in a surveillance system with decreasing sensitivity, would result in a change in the relative distribution of serotypes. There was no evidence of an increase in Hia relative to other non-b or non-typeable isolates from pre- to post-vaccination periods.

Evidence of improving social determinants of health that may have led to lower rates of infectious diseases include a decline in infant and child mortality in the past 20 years, and an increase in school retention rates and an improvement in housing for Indigenous compared to non-Indigenous people (1). However, substantial disparities continue with respect to the social determinants of health and many health indicators including rates of respiratory disease, particularly in remote areas (1).

False-positive Hi diagnoses by serum agglutination assay using CDC or Difco antisera have been identified, in comparison to PCR, in particular for serotype a (16). However, the applicability of this to the current study is unclear as the kits and antisera evaluated by Satola et al. were not in use in the laboratories involved in this study. False-positives are unlikely to be prominent in the post-vaccination Australian data, as PCR confirmation was frequently conducted at ICPMR, false-positive rates were found to be negligible, and the same monovalent a, c–f antisera were used at IMVS. The possibility of false-positive results in the pre-vaccine study from the NT cannot be discounted, although a very similar serotype distribution was found in a population of NT Indigenous children from a similar period tested in another state (17). As more precise typing methods such as pulsed-field gel electrophoresis or gene sequence typing are not available, it is not possible to comment on clonal distribution of Hi isolates from Australian samples.

The average annual incidence rate of Hia in NT and SA Indigenous children aged <5 years from 2001 to 2011 (11/100,000) is similar to that reported in the Navajo and Alaskan Native peoples but appears to be lower than that reported from northern Canada (Table II). Differences in surveillance and testing practices cannot be discounted as explanations for differences in rates.

**Table II:** Hia invasive disease rates in Indigenous children in the post-Hib vaccine era

Author	Indigenous population	Years	Cases per 100,000 population per year
Bruce (7)	Alaskan Native <2 years	2000–2005	21
Bruce (7)	North Canadian <2 years	2000–2005	102
McConnell (18)	Nunavut <5 years	1996–2001	70
McConnell (18)	Western Provinces <5 years	1996–2001	4
Millar (19)	Navajo <5 years	1988–2003	20
Menzies (current study)	Australian Aboriginal <5 years	2001–2011	11

## Conclusion

There is no evidence of increases in invasive disease due to Hia, other specific non-b types, or non-typeable Hi in Australian Indigenous children. These data suggest that the increase in Hia sometime after the introduction of Hib vaccine, as seen in the North American Arctic Region, is not common to all populations with high pre-vaccine rates of invasive Hib disease. However, small case numbers and the lack of molecular subtyping and PCR confirmation of pre-vaccine results complicate comparisons with North American epidemiology.
